# Salivary estradiol and progesterone across the menstrual cycle: Association and agreement with serum concentrations

**DOI:** 10.14814/phy2.71026

**Published:** 2026-07-28

**Authors:** Samantha J. Goldenstein, Emily E. Bechke, Sandra J. Shultz, Catherine McDowell, Laurie Wideman

**Affiliations:** ^1^ Department of Kinesiology University of North Carolina‐Greensboro Greensboro North Carolina USA

**Keywords:** menstrual function, physically active female, saliva, serum, sex steroid hormones

## Abstract

Saliva provides a noninvasive alternative to serum for serial sampling of estradiol and progesterone. This study examined salivary estradiol and progesterone patterns and the relationship between salivary and serum estradiol, progesterone, and the estradiol to progesterone ratio (E2:P) across the menstrual cycle (MC) in physically active females. Saliva was collected daily from females over one or two MCs, with blood collected five times across two MCs. At‐home ovulation tests and a retroactive serum progesterone (≥5 ng·mL^−1^) identified ovulation. Salivary estradiol and progesterone profiles followed expected serum patterns. Repeated measures correlations (*r*_rm_) showed strong significant associations between salivary and serum progesterone (*r*_rm_ = 0.58, *p* < 0.001) and salivary and serum E2:P (*r*_rm_ = 0.57, *p* < 0.001), but not salivary and serum estradiol (*p* = 0.09). No associations emerged in MCs with serum progesterone <5 ng·mL^−1^. Linear mixed models confirmed positive associations for serum and salivary progesterone (*β* = 0.02, *p* < 0.001) and E2:P (*β* = 9.44, *p* < 0.001). Bland–Altman analyses indicated poor agreement between salivary and serum concentrations. Although saliva does not directly replace serum measures, it may better reflect the biologically active hormone fraction and offers a practical tool for tracking hormonal patterns.

## 1 INTRODUCTION

Females are under‐studied in research, with estimates of only 5%–14% of studies across all disciplines examining outcomes by sex (Abbasi, 2023). The challenge of controlling for and measuring hormonal variations across the menstrual cycle is cited as the main barrier for female inclusion (Wilson et al., 2020). To avoid the complexity of the menstrual cycle, many researchers perform testing in the early follicular phase, when ovarian sex steroid hormones (i.e., estradiol, progesterone) are the lowest. However, females spend less than 20% of their reproductive years in the early follicular phase and although logistically easier, this design limits the validity of findings (Stanhewicz & Wong, 2020). Importantly, in premenopausal females, sex steroid hormones are major determinants of female health status, highlighting the need to assess multiple timepoints within a menstrual cycle. Therefore, providing noninvasive, accessible and valid methods to measure sex steroid hormones across the menstrual cycle would reduce barriers to female inclusion in research.

Sex steroid hormones measured in serum are considered the gold standard and free (i.e., bioavailable only) or total (i.e., bioavailable plus bound) hormones may be measured at a single point‐in‐time. However, multiple serum measurements are costly, invasive, require travel to facilities, and can cause participant discomfort and anxiety. On the other hand, saliva can be self‐collected, stored in a home freezer for up to a month without degradation of hormones, and later delivered for analysis (Bellem et al., 2011). While only free hormone levels are passively diffused into saliva, resulting in 1–2% of estradiol and progesterone serum concentrations (Huang et al., 2023), measuring the bioavailable hormones may be a better indicator of hormone exposure than total hormones (Bikle, 2021). Saliva‐based hormone quantification offers several other advantages, including reduced participant burden, minimal invasiveness, and the convenience of field‐based sampling.

Previous studies publishing results from pooled saliva and serum report significant correlations from 0.60 to 0.93 for estradiol (Shirtcliff et al., 2000; Wong et al., 1990) and 0.75–0.93 for progesterone (Choe et al., 1983; Ellison, 1993; Vuorento et al., 1989; Wong et al., 1990) in premenopausal females. However, in populations with low estradiol and progesterone, nonsignificant correlations of −0.07 and 0.32 were observed in males and postmenopausal women not on hormone replacement therapy, respectively (Shirtcliff et al., 2000; Tivis et al., 2005). In these prior studies, saliva was measured using radioimmunoassays (RIA), which require specialized equipment, expose staff to radiation hazards, require handling and disposing of radioactive waste (Suresh et al., 2011), and therefore, are not feasible for all researchers. Commercially available enzyme‐linked immunosorbent assays (ELISA) for salivary assessment of estradiol and progesterone are relatively simple to execute, cost effective, and highly sensitive, but have yet to be validated in human menstrual cycle research, particularly when circulating hormone levels are low.

It is well‐established that exercising and sedentary females have similar hormonal patterns (e.g., increased luteal progesterone) across ovulatory menstrual cycles, yet exercising females experience lower sex steroid hormone concentrations compared to sedentary females (De Souza et al., 2003; Fisher et al., 1986; Jasienska et al., 2006; Matthews et al., 2012; Stoddard et al., 2006). Furthermore, physically active females exhibit a higher prevalence of menstrual dysfunction, which often includes either erratic or suppressed sex steroid hormone levels. To better understand these differences and the impact on females, alternative research methods to serum must be evaluated. To utilize less invasive methods, such as saliva, to track estradiol and progesterone across time, it is important to know if the sensitivity of ELISAs used to assess salivary samples is low enough to quantify estradiol and progesterone concentrations in premenopausal physically active females with hormonal concentrations at or below the lower end of normative ranges. Therefore, the purpose of this study was to describe salivary estradiol and progesterone profiles across the menstrual cycle and to evaluate the association and agreement between salivary and serum estradiol, progesterone, and the estradiol to progesterone ratio (E2:P) at multiple timepoints across the menstrual cycle in physically active females. We hypothesized that both salivary estradiol and progesterone profiles would follow the expected patterns consistent with serum profiles. We further hypothesized that salivary and serum estradiol, progesterone, and E2:P would be positively associated and show good agreement across the menstrual cycle.

## 2 METHODS

### 2.1 Participants and study design

Healthy, 18–35‐year‐old females with normal length menstrual cycles (21–35 days) were recruited from the University of North Carolina‐Greensboro via flyers on campus, undergraduate classes, and emails to the campus dormitory residents as well as the surrounding metropolitan area. Screening procedures consisted of questions regarding menstrual cycle history, exercise, and medical history via a health history questionnaire for overall health. Inclusion criteria were as follows: (1) engage in a minimum of 2.5 h of exercise per week spread across at least 3 days for six consecutive months, (2) free from metabolic or cardiovascular diseases, eating disorders, polycystic ovarian syndrome, (3) absence of hormonal contraceptive use for at least 3‐months and not taking medications that alter the metabolic or reproductive hormones (e.g., anxiety, depression, stimulants, daily use of nonsteroidal anti‐inflammatory drug), (4) not using tobacco products (e.g., smoking, vaping), (5) not actively dieting to lose weight, and (6) not pregnant or plans to become pregnant during the duration of the study.

**FIGURE 1:**
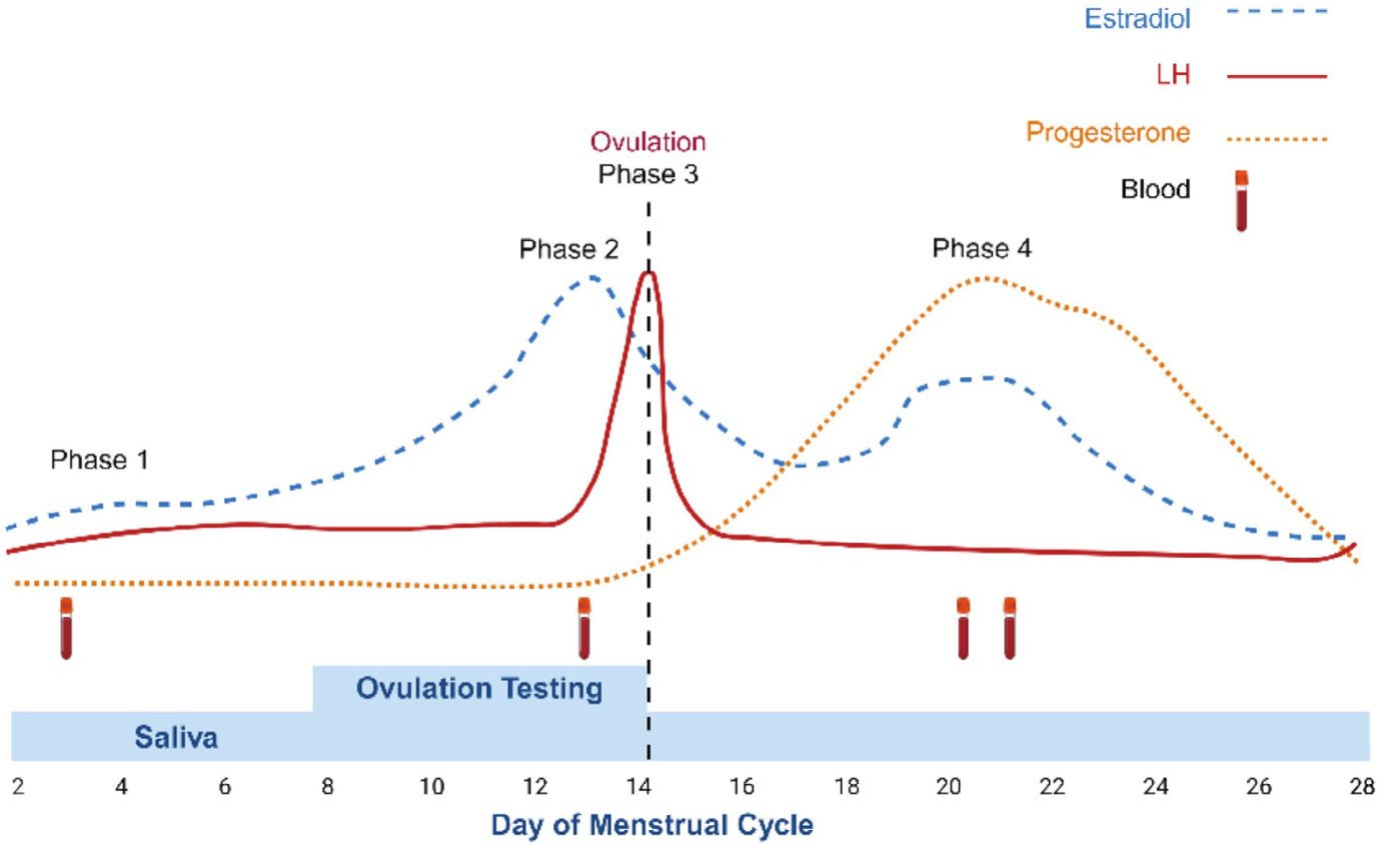
Research study design for an idealized 28‐day menstrual cycle one (MC1). Cycles were consecutive but did not have to occur in the same order between participants. MC2 (not shown) consisted of daily saliva collection, ovulation testing, and one serum collection in Phase 4. LH, luteinizing hormone. Figure created with BioRender.

The study assessed participants across two consecutive menstrual cycles (MC), with different requirements per cycle (Figure 1, shows MC1 only). The study began on the first day of menses (Day 1). Saliva collection occurred daily. Serum was collected at five different timepoints across two menstrual cycles (MC1 and MC2) to capture key phases (described below) of the menstrual cycle (Elliott‐Sale et al., 2021). Within MC1, the first visit (Phase 1) consisted of the collection of saliva, serum, and anthropometric measures. Three additional visits took place during MC1 to collect serum: one in Phase 2 and two in Phase 4. Additionally, in MC2, serum was collected on a single day in Phase 4. No serum sampling was conducted during Phase 3 due to logistical constraints and participant burden. Each visit took place between 0500 and 1000 h and participants fasted for at least 8 h prior to the visit. All study protocols and procedures were approved by the University of North Carolina‐Greensboro Institutional Review Board (IRB #FY23‐36) and all participants provided written informed consent prior to enrollment in the study.

Serum collection occurred during Phase 1, Phase 2, and Phase 4 of the menstrual cycle as recommended by Elliot Sale et al. (Elliott‐Sale et al., 2021). Phase 1 occurred during menses (i.e., early follicular) when estradiol and progesterone should be low and this visit occurred between Day 2 and 5 of the MC. Phase 2 (i.e., late follicular) captured rising estradiol, with the serum collection day determined by cycle length and estimated ovulation day (Soumpasis et al., 2020) (see Table 1, shows ovulation testing days only). Since daily serum sampling was not part of the study design, the Phase 2 serum collection was scheduled based on the estimated timing of the estradiol peak given the normative data compiled by Soumpasis et al. (2020). Thus, if a positive ovulation test was confirmed before the scheduled Phase 2 serum collection, the Phase 2 blood draw was omitted. At home ovulation tests detect the luteinizing hormone (LH) surge for Phase 3, and thus, the presumed day of ovulation. Phase 4 captured high estradiol and progesterone after a presumed positive ovulation (i.e., Phase 3) and serum was collected twice within 5–8 days following a positive ovulation test in MC1 and once in MC2. Presumed anovulatory cycles were still included in the Phase 4 timepoint even though a rise in estradiol and progesterone may not have occurred. Therefore, if a positive ovulation test was not achieved, the Phase 4 visit was scheduled within 2 days of the final day of ovulation testing as outlined by Table 1.

**TABLE 1:** Ovulation testing days based on menstrual cycle length.

Menstrual cycle length (days)	Ovulation testing days
21–23	5–18
24–28	7–20
29–35	8–25

*Note*: Testing days are the number of days following the onset of menses (day 1).

### 2.2 Study procedures

#### 2.2.1 Anthropometrics

Nude body mass was measured to the nearest 0.1 kg on a digital scale (WB‐800S Plus; Tanita Corporation, Tokyo, Japan) and height was measured by a wall mounted stadiometer (Model216; Seca, Chio, CA) to the nearest 0.5 cm at the beginning of the study. Body mass index (BMI) was calculated as body mass divided by height squared (kg·m^−2^).

#### 2.2.2 Ovulation testing

Participants used at home ovulation test strips to assess a rise in LH (Pregmate, Hollywood, FL) in both menstrual cycles. Ovulation testing days were based on the self‐reported length of the previous menstrual cycle (Table 1) and days were selected as the highest probability of ovulation occurring within that menstrual cycle based on previous research (Soumpasis et al., 2020). Each day participants sent a picture of that day's ovulation test to the research team via WhatsApp (encrypted data) and continued daily testing until the research team confirmed a positive ovulation test or the number of testing days had been reached (see Table 1). If participants failed to send a picture for two days, the research team contacted the participant. At home ovulation testing is proven to be accurate, but ovulation was also confirmed retroactively by serum progesterone values (Janse De Jonge et al., 2019; Leiva et al., 2014).

#### 2.2.3 Menstrual cycle characteristics and classification

A menstrual cycle was defined as the first day of menses (day 1) until the day prior to onset of the next menses. Failure to obtain a (1) positive ovulation test and (2) serum progesterone ≥5.0 ng·mL^−1^ resulted in the cycle being presumed as anovulatory (Bakdash & Marusich, 2017). Eumenorrheic cycles are defined as presumed ovulatory cycles with a menstrual length between 21 and 35 days. Although ovulation was confirmed with two methods, transvaginal ultrasound is the only definitive way to determine ovulation. Given this, we chose to label this variable as presumed ovulation.

#### 2.2.4 Saliva collection and analysis

Saliva was collected daily with pre‐labeled polyethylene storage tubes and straws to aid the collection process. Participants were instructed to collect saliva via passive drool immediately after waking and before eating, drinking, or brushing their teeth. Saliva samples were immediately stored in a home freezer (−20°C) until meeting with a research team member on the designated days (Figure 1), then stored at −80°C until analyses.

Free salivary 17‐β estradiol (Salimetrics: 1‐3702) and progesterone (Salimetrics: 1‐1502) were assayed by commercially available enzyme‐linked immunoassay (Salimetrics‐ America, State College, PA). Saliva samples were thawed at room temperature and then centrifuged at 13,000 rpm for 15 min prior to assay. Samples were run in duplicate and reanalyzed if the intra‐replicate coefficients of variation were >25%. The sensitivity of salivary estradiol and progesterone assays are <0.1 pg·mL^−1^and 5 pg·mL^−1^, respectively.

#### 2.2.5 Serum collection and analysis

Blood samples were collected via venipuncture using universal precautions. A total of ~10 mL of venous blood was drawn into serum tubes and allotted a clot time (≥20 min) at room temperature prior to centrifugation at 4°C for 12 min at 3000 rpm. The serum was pipetted into cryovials in ~0.5 mL aliquots and stored at −80°C until analyzed.

Serum was assayed for 17‐β estradiol (IB79103) and progesterone (IB79105) by commercially available enzyme‐linked immunoassay (IBL‐ America, Minneapolis, MN). Samples were run in duplicate and reanalyzed if any of the intra‐replicate coefficients of variation were >20%. The sensitivity of serum estradiol and progesterone assays was <1.399 pg·mL^−1^ and 0.045 ng·mL^−1^, respectively. Presumed ovulation was confirmed with serum progesterone levels.

#### 2.2.6 Statistical analysis

Statistical analyses were conducted using R statistical software (v2023.03.0; R core Team, 2023) with statistical significance accepted at *p* < 0.05. To evaluate the association and agreement between salivary and serum hormone concentrations across repeated measurements, a multi‐step analytical approach was employed. First, within‐participant associations between repeated salivary and serum concentrations of estradiol, progesterone and estradiol to progesterone ratio (E2:P) were assessed using a repeated measures correlations (*r*_rm_) (rmcorr, version 0.4.1 (Bakdash & Marusich, 2017)). Each hormone was analyzed separately, regardless of menstrual function. Repeated measures coefficient magnitude was interpreted using the following ranges: 0–0.29, small; 0.30–0.49, moderate; ≥0.50, large (Bakdash & Marusich, 2017). Secondary analyses were conducted in participants with a serum progesterone ≥5 ng·mL^−1^ and then again with participants that had a serum progesterone <5 ng·mL^−1^. A sensitivity analysis was performed to examine whether removing standardized residuals from the rmcorr‐equivalent model with values exceeding ±2.5 SD influenced the results and there were no changes detected. Second, a linear mixed‐effects model was performed with the R “lme4” package (Bates et al., 2015). The linear mixed models used a fixed effect for timepoint and a random effect for participant, which accounts for within‐ and between‐participant variability. Finally, Bland–Altman analysis adapted for repeated measures assessed agreement between salivary and serum concentrations. Differences between saliva and serum were modeled using linear mixed‐effects models with participant included as a random effect, allowing estimation of the mean difference (bias) and total variance incorporating both within‐ and between‐participant variability. Limits of agreement (LOA) were calculated as the mean difference ± 1.96 times the total standard deviation. Analyses were conducted on the log transformed data and estimates of bias and LOA were back‐transformed and expressed as saliva‐to‐serum ratios where appropriate.

Descriptive data were summarized as mean ± standard deviation (SD). The results from the repeated measures correlation are reported as *r*_rm_, [95% Confidence Interval (CI)], *p*‐value, whereas linear mixed‐effects models are reported as mean difference [95% CI], *p*‐value throughout the manuscript.

## 3 RESULTS

### 3.1 Participants

A total of 26 women were recruited and consented to participate. Of the 26 women, five dropped out of the study due to: menstrual cycle irregularities (*n* = 1), pregnancy (*n* = 1), and time constraints (*n* = 3). Of the remaining 21 participants, participants were excluded if less than two timepoints with saliva and serum samples were obtained (*n* = 3). A menstrual cycle was excluded if no serum samples were obtained (*n* = 3). Thus, 18 participants with a total of 33 menstrual cycles were included in these analyses. Participants were primarily Tier 1–2, indicating a recreationally active to trained cohort (McKay et al., 2022). Participant and menstrual characteristics are displayed in Table 2.

**TABLE 2:** Participant and menstrual characteristics.

	*n* = 18
Age	21 ± 3
Height (cm)	163.0 ± 4.8
Body mass (kg)	64.3 ± 12.0
BMI (kg·m^−2^)	24.2 ± 4.1
Menstrual cycle length (days)	29 ± 2
Range of menstrual cycle length (days)	22–35
% of eumenorrheic cycles	67%

*Note*: Mean ± SD. BMI, body mass index.

### 3.2 Salivary and serum estradiol and progesterone

**FIGURE 2:**
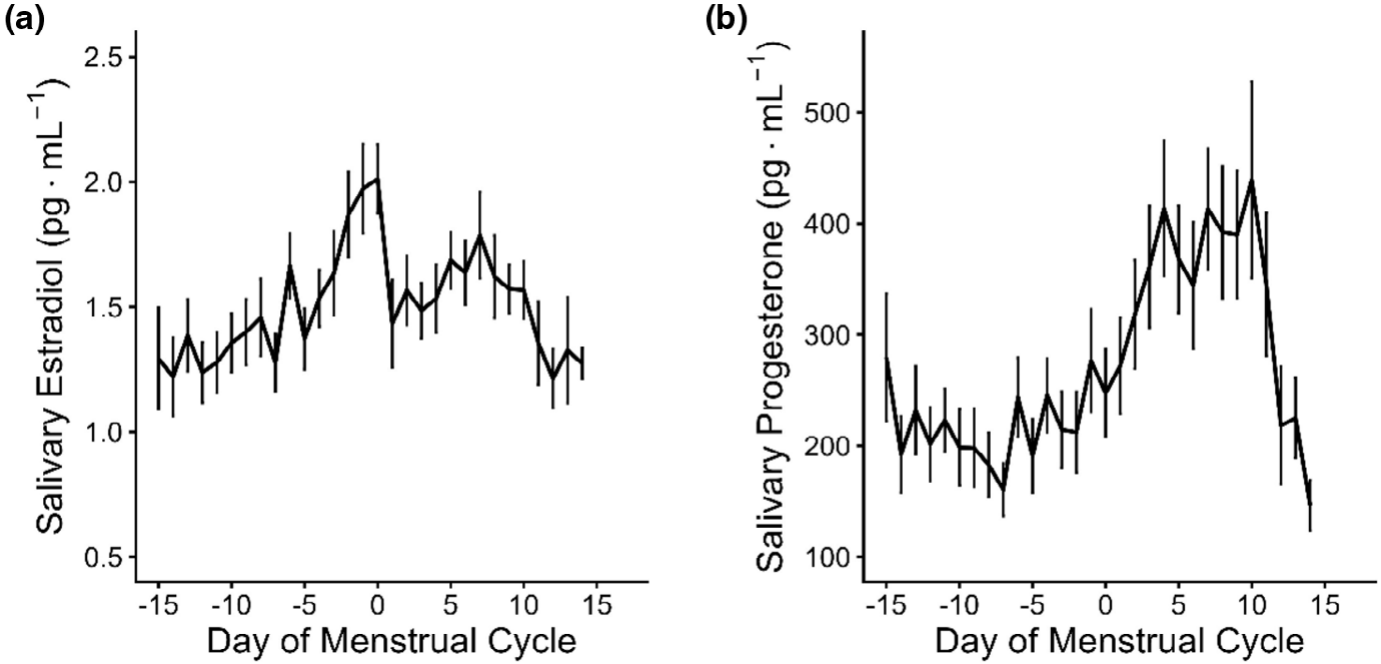
Estradiol (a) and progesterone (b) concentrations (means ± SE) in daily saliva samples across the menstrual cycle in 22 menstrual cycles centered on day of ovulation (day 0).

Salivary profiles for estradiol and progesterone for presumed ovulatory cycles (*n* = 22 cycles) are displayed in Figure 2, centered on the estimated day of ovulation (Day 0). Both estradiol and progesterone followed patterns similar to expected profiles in serum, with estradiol peaking prior to the estimated day of ovulation, and a second, smaller increase after ovulation. Progesterone remained low then increased after the estimated day of ovulation.

**FIGURE 3:**
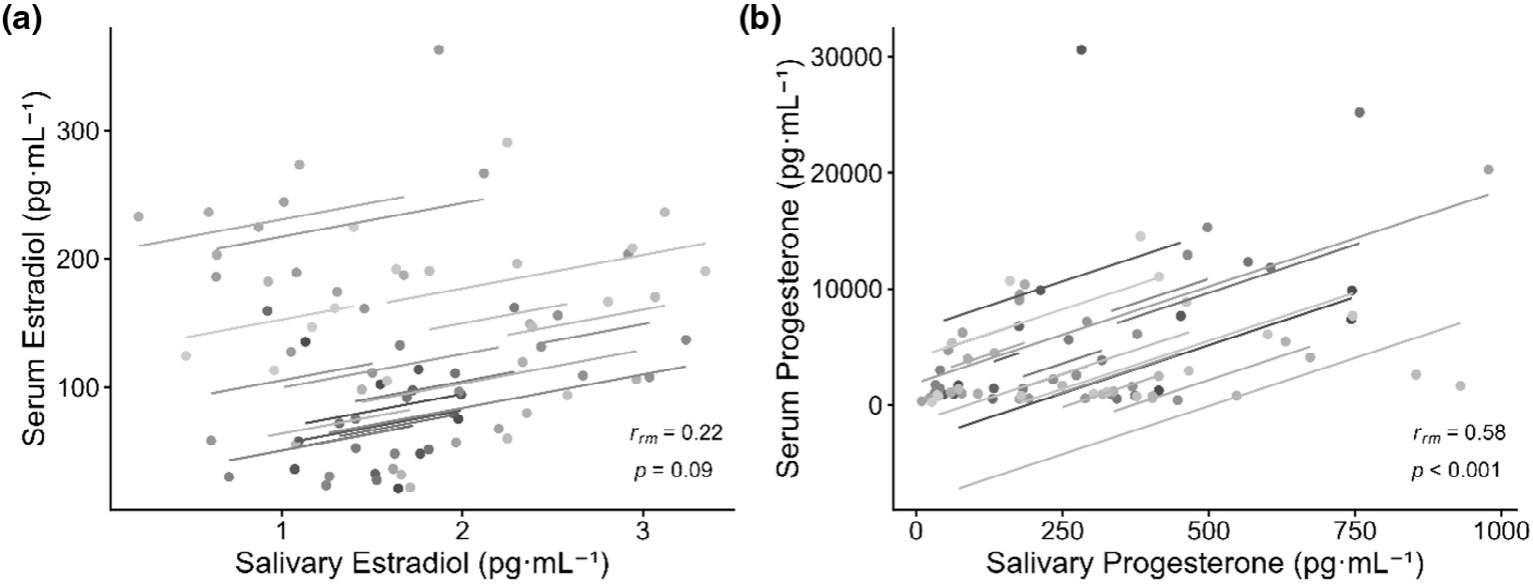
Salivary and serum repeated measures correlations for (a) estradiol, (b) progesterone. E2, estradiol; P, progesterone.

Salivary and serum estradiol (*n* = 79 paired samples), progesterone (*n* = 77 paired samples), and E2:P concentrations are presented in Table 3. This table is representative of all paired samples, regardless of menstrual function. A repeated measures correlation demonstrated that salivary and serum progesterone (*r*_rm_ = 0.58, CI [0.38–0.73], *p* < 0.001; Figure 3) and the salivary and serum ratio of E2:P (*r*_rm_ = 0.57, CI [0.31–0.69], *p* < 0.001) were strongly correlated. However, the correlation between salivary and serum estradiol was small and not significant (*r*_rm_ = 0.23, CI [−0.03–0.45], *p* = 0.09; Figure 3).

**TABLE 3:** Salivary and serum concentrations for estradiol, progesterone and E2:P.

	Phase 1	Phase 2	Phase 4
Salivary Estradiol (pg·mL^−1^)	1.40 ± 0.6	1.63 ± 0.6	1.86 ± 0.8
Serum Estradiol (pg·mL^−1^)	89.74 ± 66.7	136.65 ± 62.5	136.24 ± 74.2
Salivary Progesterone (pg·mL^−1^)	224.70 ± 159.7	173.13 ± 178.9	367.35 ± 255.3
Serum Progesterone (pg·mL^−1^)	878.94 ± 320.3	927.08 ± 401.5	7596.49 ± 6492.0
Salivary E2:P	1.21 ± 1.1	2.83 ± 3.7	1.03 ± 1.4
Serum E2:P	0.13 ± 0.1	0.19 ± 0.1	0.04 ± 0.04

*Note*: Mean ± SD. E2:P, estradiol to progesterone ratio. Values are reflective of all participants, regardless of menstrual function. All serum and saliva samples collected at the Phase 4 timepoint across both menstrual cycles were pooled.

Secondary analyses evaluated only menstrual cycles that achieved a serum progesterone ≥5 ng·mL^−1^ (presumed ovulatory cycles) and the overall results were similar, with the repeated measures correlation values increasing slightly for progesterone (*r*_rm_ = 0.65, CI [0.42–0.81], *p* < 0.001) and E2:P (*r*_rm_ = 0.69, CI [0.47–0.83], *p* < 0.001), with no change in estradiol (*r*_rm_ = 0.22, CI [−0.10–0.51], *p* = 0.17). When analyses were limited to menstrual cycles with progesterone <5 ng·mL^−1^ (*n* = 28 paired samples) no associations were found with salivary and serum progesterone (*r*_rm_ = 0.08, CI [−0.43, 0.56], *p* = 0.76), estradiol (*r*_rm_ = 0.24, CI [−0.29, 0.66], *p* = 0.37), or E2:P (*r*_rm_ = 0.29, CI [−0.24, 0.69], *p* = 0.27).

A linear mixed‐effects model showed serum estradiol did not predict salivary estradiol (*β* = 0.002, CI [−0.001–0.004], *p* = 0.09). A positive association emerged with serum progesterone and salivary progesterone (*β* = 0.019, CI [0.012–0.027], *p* < 0.001) as well as serum and salivary E2:P (*β* = 9.44, CI [6.25–12.64], *p* < 0.001).

**FIGURE 4:**
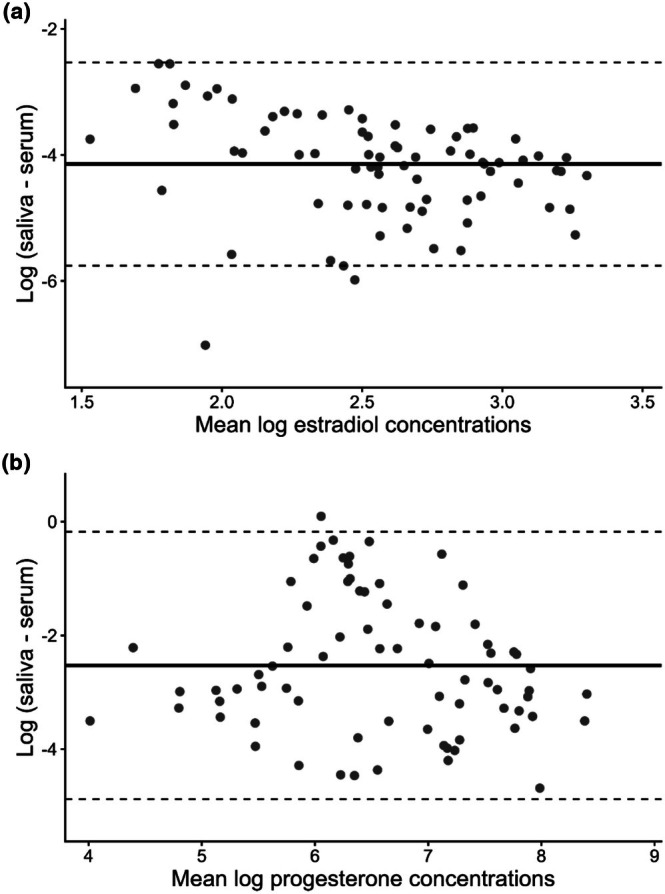
Bland–Altman plots of log‐transformed salivary and serum estradiol (a) and progesterone (b) concentrations. Solid lines indicate mean bias and dashed lines indicate the 95% limits of agreement. Back‐transformed mean bias and limits of agreement corresponded to saliva‐to‐serum ratios of 0.016 (95% LOA: 0.003–0.080) for estradiol and 0.080 (95% LOA: 0.008–0.838) for progesterone.

As expected, a log‐transformed Bland–Altman analysis demonstrated negative bias for salivary and serum concentrations of estradiol and progesterone (salivary levels were lower than serum) (Figure 4). For estradiol, the mean difference corresponded to a saliva‐to‐serum ratio of 0.016, indicating that salivary estradiol concentrations were, on average, 1.6% of serum concentrations (−98.4% relative bias). The 95% limits of agreement ranged from 0.003 to 0.080, indicating substantial variability in the saliva‐to‐serum ratio. For progesterone, the mean difference corresponded to a ratio of 0.080, indicating that salivary concentrations were approximately 8.0% of serum concentrations (−92.0% relative bias). The 95% limits of agreement ranged from 0.008 to 0.838, indicating even greater variability between methods.

## 4 DISCUSSION

This study investigated salivary hormonal patterns and the association and agreement between salivary and serum estradiol, progesterone, and E2:P in physically active females at multiple timepoints across the menstrual cycle. Importantly, when centered on day of ovulation, both salivary estradiol and progesterone followed the well‐documented hormonal patterns observed in serum across the menstrual cycle. However, only salivary progesterone and E2:P emerged with strong and significant associations to serum levels in physically active females. These findings were no longer significant when testing only anovulatory cycles (i.e., decreased hormone concentrations). Regardless of menstrual status, estradiol in saliva and serum were not significantly correlated. Furthermore, both estradiol and progesterone displayed poor agreement between saliva and serum values, indicating that these two matrices are not interchangeable when comparing absolute values.

The pattern of salivary estradiol and progesterone hormonal profiles across the menstrual cycle followed the expected and well‐documented serum profiles (Figure 2). Similarly, the serum concentrations in each phase aligned with expected normative levels (Table 3). When centered on the day of ovulation (Day 0), estradiol peaked prior to ovulation, and both estradiol and progesterone increased after presumed ovulation. Thus, if the focus of a particular research question is tracking changes in hormonal profiles across menstrual cycles, salivary samples represent a viable option; albeit, additional work would be needed to determine salivary progesterone values that discern ovulatory from anovulatory cycles. Given that salivary hormonal patterns follow the expected estradiol and progesterone profiles across the menstrual cycle, they provide little justification for the lack of correlation between serum and salivary estradiol and poor agreement in both estradiol and progesterone.

The lack of significant association between salivary and serum estradiol contradicts previous research which demonstrated a moderate to strong correlation when comparing salivary and serum estradiol (Shirtcliff et al., 2000; Wong et al., 1990). However, the research is limited, with only a few studies investigating the correlation at multiple points across the menstrual cycle and limited evidence in physically active females with potentially different menstrual function. It is well established that exercising females experience lower sex steroid hormone concentrations compared to sedentary females (De Souza et al., 2003; Fisher et al., 1986; Jasienska et al., 2006; Matthews et al., 2012; Stoddard et al., 2006) and experience a higher prevalence of menstrual dysfunction. Thus, low hormone concentrations may have contributed to the nonsignificant association. Other populations with low sex steroid hormones (i.e., men, postmenopausal females) have demonstrated poor salivary and serum correlations. In a study assessing pooled salivary and serum estradiol samples for males and females, there was no significant correlation for salivary and serum estradiol (Shirtcliff et al., 2000). Yet, when stratified by sex, male samples (with lower estradiol concentrations than females), did not show a significant association (*r* = −0.07), while the female samples were strongly correlated (*r* = 0.68) (Shirtcliff et al., 2000). A nonsignificant association between saliva and serum estradiol at lower concentrations has also been reported in postmenopausal women who were not on hormonal therapy (Tivis et al., 2005).

While both estradiol and progesterone are lipophilic molecules that passively diffuse into the saliva and are present at 1%–2% of the total serum concentrations, estradiol is present in much lower concentrations throughout the menstrual cycle compared to progesterone (Table 3). These larger progesterone concentrations are typically easier to measure and may result in stronger correlations. For instance, in the current investigation, the associations between salivary and serum progesterone were no longer evident when assessing females without presumed ovulation (i.e., serum progesterone ≤5 ng·mL^−1^). This could indicate less reliability with salivary and serum measurements at lower concentrations even though all salivary and serum samples were above the respective ELISAs lower sensitivity levels. Similarly, in a comparison of serum estradiol and progesterone measured by both automated immunoassays and gas chromatograph/mass spectrometry (GC/MS), a bias was revealed such that an increased imprecision was observed in many immunoassays as estradiol and progesterone concentrations decreased and overall, progesterone measurements demonstrated higher precision than estradiol (Coucke et al., 2007). Future research should investigate discrepancy between serum and saliva to identify if the lack of correlation is due to measurement issues or if there are physiological differences in free versus total estradiol and progesterone at these lower concentrations.

Although Lu et al. reported a correlation between salivary and serum estradiol of 0.71 with moderate to strong individual correlations when evaluating multiple measurements in six participants across a menstrual cycle, the pooled correlation was nonsignificant. Furthermore, when all participants were pooled (*N* = 7), the correlation between salivary and serum estradiol decreased (*r* = −0.08) with a wider range of individual correlations (*r* = −0.42 to 0.85) (Lu et al., 1999). This data emphasizes the intra and inter‐individual variations of hormones across the menstrual cycle and while salivary and serum estradiol had a strong correlation in some, this correlation was not significant in others. In the current study, each participant had a maximum of five paired samples and therefore we were not adequately powered to investigate the individual correlations. However, the statistical methods chosen for these analyses (i.e., repeated measures correlation) accounted for intra‐individual variation.

Another possible explanation for the nonsignificant estradiol association for serum and saliva relates to differences in physiology and methodology. Estradiol can be metabolized to >100 conjugated and unconjugated metabolites, some of which cross react with estradiol antibodies. Stanczyk et al. examined estradiol with conventional and direct (i.e., no extraction step) RIAs in postmenopausal females. Out of 14 estradiol serum samples, the direct RIA quantified lower estradiol concentrations than the conventional RIA in six samples, yet higher concentrations in eight samples. This suggests a mixed pattern of cross reactivity, although it is unclear if the cross reactivity is dependent on estradiol concentrations (Stanczyk et al., 2010). The cross reactivity for the serum and saliva estradiol ELISAs used in the current study were 6.9% and 1.3%, respectively, potentially resulting in an over estimation of estradiol in serum.

The strong correlation between salivary and serum progesterone that we observed (*r*_rm_ = 0.58) aligns with previous research, although the correlation was slightly lower than most of the previously reported values (i.e., *r* = 0.59–0.99). To our knowledge, no previous studies have investigated the E2:P association in saliva and serum even though it is an important measurement when assessing premenopausal females, as E2:P fluctuates across the menstrual cycle and can influence systems such as core body temperature and sleep (Giersch et al., 2021; Grant et al., 2020). Despite the lack of association with estradiol in saliva and serum, the salivary and serum E2:P demonstrated a strong correlation (*r*_rm_ = 0.57).

As expected, salivary estradiol and progesterone concentrations were consistently lower than serum concentrations, 1.6% and 8%, respectively. While the salivary estradiol percentage is consistent with previous research (Lu et al., 1999), the progesterone percentage is higher. Furthermore, the LOA were wide, particularly in progesterone, indicating substantial variability in the saliva and serum relationship, despite moderately strong correlations. Previous studies have reported fluctuating free progesterone concentrations across the menstrual cycle (Choe et al., 1983; Evans, 1986; Vitzthum et al., 2025), which may explain our elevated average free progesterone concentrations and wider LOA.

The Bland–Altman analysis demonstrated poor agreement between saliva and serum for estradiol and progesterone, indicating that while similar patterns were observed across the menstrual cycle, these matrices may not be interchangeable. However, these measures represent different hormone states. Serum measures represent total hormone levels (i.e., free and bound) while salivary concentrations reflect the free, unbound fraction that is biologically active. As such, saliva may provide a better indicator of hormone exposure than total hormones (Bikle, 2021).

The free hormone hypothesis states that the free hormones will enter cells and exert biological effects and therefore free hormone levels are a better reflection of the hormonal state than total hormone concentrations (Bikle, 2021). Both sex steroid hormones bind loosely to albumin but also bind with high affinity to separate proteins, thereby regulating the proportion of free hormone in circulation differently. Sex hormone binding globulin (SHBG) binds estradiol while progesterone binds to corticosteroid‐binding globulin (CBG) (Hammond, 2016). SHBG is allosteric, with binding at one site altering the *K*_a_ to the other SHBG binding site; therefore, potentially influencing estradiol binding and free estradiol concentrations (Rosner, 2015). SHBG concentrations are influenced by the concentrations of sex steroids, typically increasing with increased estradiol and decreasing with increased testosterone (Mean et al., 1977). As changes in estradiol and SHBG concentrations take place, the *K*_d_ also fluctuates, which can result in nonlinear binding of estradiol to SHBG and indicates a concentration‐dependent allosteric interaction on the SHBG binding sites (Jasuja et al., 2021). Furthermore, dihydrotestosterone (DHT) and testosterone both exhibit greater binding capacity to SHBG than estradiol (Mean et al., 1977; Rosner, 2015). If DHT or testosterone are elevated, this could result in increased free estradiol and alter the free and total correlation. Changes in free and total hormones occur with many hormone disorders or physiological states. For example, polyendocrine metabolic ovarian syndrome (PMOS) presents with elevated free estradiol and decreased SHBG (Lobo et al., 1981), while the proportion of progesterone bound to CBG increases during pregnancy (Pugeat et al., 1981), further indicating that free hormones measurements are needed in addition to total concentrations.

This study focused on physically active females, who typically have lower hormone concentrations than sedentary females, to determine the extent to which saliva concentrations were correlated with serum. The strength of this study is the focus on physically active females, a rigorous daily collection schedule for saliva across two menstrual cycles and adherence to strict inclusion criteria that provided a homogenous sample of females from a physical activity perspective (normative activity levels, not elite athletes). However, the present study is not without limitations. To minimize participant burden, correlations with serum were limited to five carefully selected timepoints across the menstrual cycle with the intent of capturing substantial variability in the hormone levels. It is difficult to know if stronger relationships would emerge with more paired time points, but future studies should consider a more fine‐grained analysis. Furthermore, due to the variability in menstrual cycles in our participants, it was not always possible to collect all five planned blood samples; a caveat that needs to be considered when planning future studies.

In conclusion, this study demonstrated that salivary estradiol and progesterone profiles follow similar patterns to serum. However, only progesterone and E2:P salivary samples, not estradiol, are strongly associated with serum concentrations in eumenorrheic physically active females, whereas these correlations were absent with presumed anovulatory cycles. These findings indicate that salivary estradiol, progesterone and E2:P may provide a promising alternative to serum if the major focus of the research is assessing and/or tracking hormonal patterns across the menstrual cycle. However, despite moderately strong correlations between salivary and serum progesterone, the Bland–Altman analysis revealed poor agreement between saliva and serum for both estradiol and progesterone, suggesting that salivary measures are not systematically reflective of serum concentrations and thus, are not direct substitutes for serum concentrations. Future research should identify contexts in which saliva provides greater clinical or physiological relevance than serum, particularly for monitoring dynamic hormonal changes, aim to recruit larger sample sizes that include females with menstrual dysfunctions beyond anovulatory cycles (i.e., luteal phase defects), consider statistical methods that account for physiologically‐relevant ‘lags’ between hormonal levels in saliva and serum to assess effects on correlation strength and increase the number of matched samples for saliva and serum across the menstrual cycle. Since current research suggests there is considerable interindividual, as well as menstrual cycle‐dependent variability in the saliva‐serum patterns for estradiol and progesterone, future work should attempt to describe these nuanced profiles and the utility of each for specific types of female‐focused research.

## AUTHOR CONTRIBUTIONS

**Samantha J. Goldenstein:** Conceptualization; data curation; formal analysis; investigation; methodology; project administration; visualization. **Emily E. Bechke:** Conceptualization; data curation; formal analysis; investigation; methodology; project administration. **Sandra J. Shultz:** Formal analysis; methodology; validation. **Catherine McDowell:** Data curation; project administration. **Laurie Wideman:** Conceptualization; formal analysis; funding acquisition; investigation; methodology; supervision; validation.

## FUNDING INFORMATION

Open Access funding provided by the University of North Carolina at Greensboro.

## CONFLICT OF INTEREST STATEMENT

All authors declare no conflicts of interest, financial or otherwise.

## ETHICS STATEMENT

All study protocols and procedures were approved by the University of North Carolina‐Greensboro Institutional Review Board (IRB #FY23‐36).

## CONSENT

All participants provided written informed consent prior to enrollment in the study.

## Data Availability

Data generated or analyzed during this study are available from the corresponding author upon reasonable request.
